# Cu or Fe‐Exchanged Natural Clinoptilolite as Sustainable Light‐Assisted Catalyst for Water Disinfection at Near Neutral pH

**DOI:** 10.1002/cplu.202500225

**Published:** 2025-09-18

**Authors:** Paula Prieto‐Laria, Pilar Fernández‐Ibáñez, A. Rabdel Ruiz‐Salvador, Inés Canosa, Amando Flores, Carlos Salameh, José Enrique Domínguez‐Santos, Nuria Ofelia Núñez, Menta Ballesteros, Tania Farías

**Affiliations:** ^1^ Departamento de Biología Molecular e Ingeniería Bioquímica Universidad Pablo de Olavide Ctra. Utrera km. 1 Sevilla 41013 Spain; ^2^ Nanotechnology and Integrated BioEngineering Centre Ulster University Belfast BT15 1AP UK; ^3^ Departamento de Sistemas Físicos Químicos y Naturales Universidad Pablo de Olavide Ctra. Utrera km. 1 Sevilla 41013 Spain; ^4^ Centro de Nanociencia y Tecnologías Sostenibles (CNATS) Universidad Pablo de Olavide Ctra. Utrera km. 1 Sevilla 41013 Spain; ^5^ Centro Andaluz de Biología del Desarrollo CSIC/Universidad Pablo de Olavide/Junta de Andalucía Ctra. Utrera km. 1 Sevilla 41013 Spain; ^6^ Instituto de Ciencia de Materiales de Sevilla (CSIC‐US) C/Américo Vespucio, 49 Sevilla Spain; ^7^ División de Química y Tecnología de Materiales Instituto de Ciencia y Tecnología de Materiales (IMRE) Universidad de La Habana Zapata y G La Habana 10400 Habana Cuba

**Keywords:** bacteria inactivation, heterogeneous photocatalysts, iron and copper exchange, natural zeolite, visible light

## Abstract

Natural zeolites can be used to obtain effective catalysts for heterogeneous photocatalytic reactions due to their low cost and favorable physicochemical properties for water treatment. In this work, a natural clinoptilolite is modified by incorporating iron (NZ–Fe) and copper (NZ–Cu) as compensation cations through ion exchange processes. Metals incorporation and structural stability are demonstrated through X‐ray diffraction, Fourier transform infrared spectroscopy, and scanning electron microscopy. DR‐UV–Vis measurements are used to estimate the bandgap and predict the photocatalytic performance of both materials. Their effectiviness in heterogeneous photocatalytic systems is confirmed by evaluating the inactivation of *E. coli* as a model pathogen in water. The bacterial detection limit (initial ≈10^6^ CFU/mL) is reached using 1 gL^−1^ of both catalysts, 100 ppm of H_2_O_2_ under visible light (410–710 nm) and near neutral pH in 2 h, with no post‐treatment regrowth observed. Experimental data are analyzed according to the Chick–Watson, Weibull, and Hom disinfection kinetic models. Although more hydroxyl radicals are generated (trapping tests) and less iron leachate is observed for NZ–Fe, good reusability is attained for three disinfection cycles when NZ–Cu is used. This makes copper‐exchanged clinoptilolite a suitable and low‐cost photocatalyst for water disinfection through heterogeneous photo‐Fenton‐type processes.

## Graphical abstract

Fe and Cu exchanged natural clinoptilolites are evaluated as heterogeneous photo‐Fenton catalysts for water disinfection under visible light, using *E. coli* as a model pathogen. Bacterial detection limit is reached in 2 h, with no regrowth observed. The kinetics of the reaction, the hydroxyl radicals generation, and the reuse of the catalysts are studied. Faster kinetics and better reusability is found in Cu‐ than in Fe‐clinoptilolite…

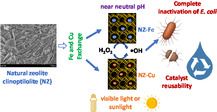

## 1 Introduction

Microporous materials have a significant presence in solving relevant problems in a wide range of applications. With regard to water, there is a long experience in using zeolites and metal‐organic frameworks for ion‐exchange and adsorption applications,^[^ ^1^, ^2^, ^3^ ^]^ thanks to their fascinating structures. In contrast, these materials have been used much less frequently in the context of water contamination with pathogens. However, this is an area that calls urgent attention as it is a significant problem that affects millions of people worldwide. According to data from the UN and WHO, ≈2.2 billion people lack access to safe drinking water, which is directly associated with the proliferation of waterborne diseases such as cholera, diarrhea, and typhoid fever.^[^ ^4^ ^]^ The UN has recognized the importance of addressing this problem by establishing access to clean water and sanitation as one of the sustainable development goals. The presence of pathogens in water is a key factor in perpetuating this global health crisis, which requires urgent measures to ensure that all individuals have access to clean and safe water. This would significantly enhance public health and well‐being worldwide. In developing countries, water stress is closely linked to water scarcity and the degradation of its quality resulting from urban development and agriculture. Overall, the reuse of treated water has been well accepted as an imperative solution to address the existing challenges.^[^ ^5^, ^6^, ^7^ ^]^

To achieve effective wastewater treatment, advanced methodologies are needed to surpass classical treatments performed in urban wastewater treatment plants, which have been proven to be inefficient for certain emerging contaminants.^[^ ^8^ ^]^ Slow release of bactericidal metals has been one of the options considered in the palestra. Rivera–Garza et al*.* showed that silver released from a natural clinoptilolite accounts for bactericidal effect in the range from 6 to 24 h of contact with bacterial cultures, depending on the initial silver content in the zeolite.^[^ ^9^ ^]^ Milán et al. demonstrated that a natural clinoptilolite packed in a column with circulating infected water achieves a 30–50% bacterial removal after 6 h of contact.^[^ ^10^ ^]^ Milenkovic et al*.* prepared a natural and a synthetic zeolite loaded with Zn, Cu, and Ag and observed that the release of silver was responsible for the bactericidal effect against *E. coli*, while the small release of copper and zinc suggested some intrinsic bactericidal effect of the zeolite itself.^[^ ^11^ ^]^ Large‐scale water treatment applications demand advanced oxidation processes (AOPs), which are known as alternative methods because of their ability to eliminate a wide range of contaminants, including persistent organic compounds, heavy metals, and pathogens in water.^[^ ^12^, ^13^ ^]^ The photo‐Fenton process stands out as one of the most effective AOPs. Its uniqueness lies in the generation of highly reactive hydroxyl radicals through a photochemical reaction driven by ultraviolet radiation or sunlight, using only hydrogen peroxide and an iron source.^[^ ^14^ ^]^ Heterogeneous photo‐Fenton treatment is a variant that involves immobilizing photoactive iron species on a solid such as zeolites and silicas, among others,^[^ ^15^ ^]^ rather than using its soluble form (homogeneous photo‐Fenton). This allows for greater catalyst reusability and efficiency, along with extended lifespan compared to homogeneous systems. Furthermore, it reduces the generation of ferrous sludge, which requires subsequent treatment and proper disposal, as seen in homogeneous photo‐Fenton treatment.^[^ ^16^ ^]^ Consequently, the heterogeneous photo‐Fenton process has recently emerged as a promising approach for removing pathogens and other contaminants from wastewater.^[^ ^15^, ^17^ ^]^ The main disadvantages of the heterogeneous photo‐Fenton process relate to selecting the appropriate solid catalyst and adapting it to water conditions. With certain catalysts, reactivity may be lower, potentially requiring longer residence times. Additionally, the synthesis and preparation of solid catalysts is often a more complex and costly process. One way to address these challenges is to use solid catalysts derived from zeolites, as well as other materials such as clays, carbon nanotubes, fullerenes, graphene, and chitosan, which have also demonstrated antibacterial activity.^[^ ^15^ ^,^ ^18^, ^19^, ^20^, ^21^ ^]^

Zeolites can be excellent heterogeneous photo‐Fenton catalysts for several reasons: 1) They possess a highly ordered 3D microporous structure, which provides them a large surface area containing active sites for the suitable diffusion of contaminants, thus improving the process's efficiency.^[^ ^22^ ^]^ 2) They can exchange metal ions, such as iron, within their structure. This enables the immobilization of iron ions in the zeolite, serving as catalysts in the photo‐Fenton process.^[^ ^23^ ^]^ 3) Zeolites are also well‐known for their chemical stability under a wide range of conditions, making them suitable as both intrinsic catalysts and supports for catalysts in environmental applications.^[^ ^24^ ^]^ This stability ensures that zeolitic catalysts are durable and do not disintegrate during the water treatment process. 4) They can be easily regenerated and reused after the photo‐Fenton process, reducing costs and minimizing waste generation, rendering them an essential tool for addressing water pollution challenges worldwide.

Synthetic zeolites are widely used in heterogeneous Fenton processes, either as supports or due to their intrinsic photochemical properties.^[^ ^20^ ^,^ ^25^, ^26^, ^27^ ^]^ However, their high cost is usually a handicap for their large‐scale usage. Using natural zeolites as catalysts in heterogeneous photo‐Fenton processes offers significant sustainability advantages. Moreover, natural zeolites possess the same beneficial physicochemical properties as synthetic zeolites,^[^ ^15^, ^28^ ^]^ when the pore size is not a restriction, since most natural zeolites have small pores. Implementing natural materials in this context not only optimizes the process of contaminant removal from water but also promotes more eco‐friendly and economical environmental treatment practices. Fukuchi et al*.* demonstrated that Fe‐natural clinoptilolite was not active per se for decontaminating 2,4,6‐tribromophenol via a Fenton process, while the addition of reducing agents such as ascorbic acid or hydroxylamine did achieve complete degradation.^[^ ^28^ ^]^ Recently, heavily dealuminated hierarchical natural clinoptilolite exchanged with iron has been used as a heterogeneous Fenton catalyst to treat biologically treated domestic sewage to remove diclofenac and ranitidine, but not simvastatin.^[^ ^29^ ^]^ Due to the extensive dealumination and the presence of mesopores, this modified clinoptilolite contains iron as exchangeable cations, as well as forming oxide nanoparticles in the mesoporosity. These nanoparticles have been shown to play an active role in the Fenton process. The inability of the catalyst to remove simvastatin was explained by the inaccessibility of the molecule to the microporosity due to its size. It should be noted that although diclofenac and ranitidine are smaller than simvastatin, they are still large enough to enter the clinoptilolite microporosity and may only access the created mesoporosity. Phan et al*.* prepared a material by calcining a mixture of natural clinoptilolite and lanthanum iron oxide (at 15, 30, and 60 wt.%) at 700 °C for photo‐Fenton degradation of rhodamine.^[^ ^30^ ^]^ Clinoptilolite without LaFeO_3_ adsorbed 30% of the dye in the dark, while a further 10% was removed by light exposure. In contrast, the composites showed marginal rhodamine adsorption and degraded the dye in light to a similar extent to the oxide, indicating that the photocatalytic activity was not provided by the clinoptilolite.^[^ ^30^ ^]^

The literature cited above encourages research into using natural clinoptilolite as a catalyst for photo‐Fenton water disinfection. However, there are several open questions that need to be addressed: Would ion‐exchanged clinoptilolite (with appropriate extra‐framework cations) be photoactive? Would ion‐exchanged clinoptilolite be sufficient for photo‐Fenton processes, or would other catalysts also be required? If metal‐exchanged clinoptilolite showed photoactivity in generating ^•^OH radicals from H_2_O_2_, would a sufficient quantity be produced in the required timeframe for disinfection? If disinfection is achieved, is bacterial inactivation effective, or does regrowth occur after treatment? Can metal‐exchanged clinoptilolite be reused in disinfection processes? It should be noted that, to our knowledge, natural zeolites have not been previously used to inactivate pathogens in water by heterogeneous photo‐Fenton systems.

In the present work, heterogeneous catalysts based on a natural clinoptilolite exchanged with iron and copper cations were evaluated for water disinfection using *E. coli* as a model bacterium by photo‐Fenton under visible light. The characterization of the materials was carried out to confirm the incorporation of iron and copper, as well as the structural stability of the modified zeolites. DR‐UV–vis measurements were used to determine the bandgap and predict the photocatalytic performance of the materials. Their effectiveness was evaluated through experiments on the inactivation of *E. coli* in water. The kinetics of the reaction and the generation of hydroxyl radicals during the reaction were analyzed, as well as the reusability of the catalysts.

## 2 Results and Discussion

### 2.1 Characterization of NZ, NZ–Fe, and NZ–Cu

**Figure 1:**
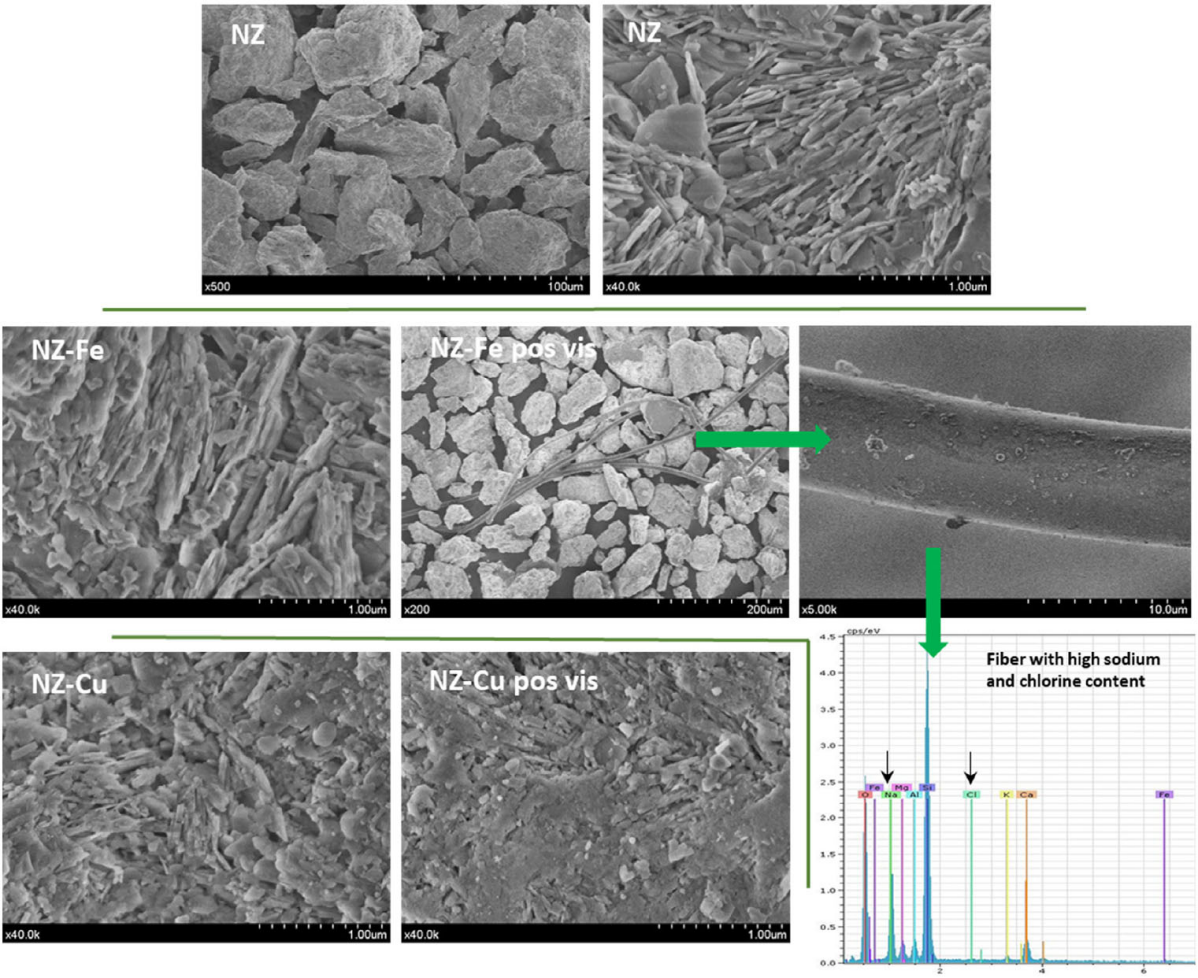
SEM micrographs of the natural zeolite and the samples enriched in iron and copper before and after photo‐fenton assays.

The NZ sample shows lamellar crystals with smooth edges that are stacked within the mineral, forming compact aggregates of about 90 µm, as can be observed in the scanning electron microscopy (SEM) images (**Figure** **1**). No significant morphological changes are observed in the prepared NZ–Fe and NZ–Cu samples. The zeolite morphology also remains unchanged after the photo‐Fenton tests, although elongated particles containing mostly sodium and chlorine appear due to the reaction medium used in the disinfection assays (NaCl 0.9%). As expected, no iron or copper oxide or hydroxide particles were found in the micrographs, since the method used ensures the exchange of both cations in natural clinoptilolite rather than the precipitation of other phases.^[^ ^31^ ^]^ The elemental chemical composition confirmed that the percentage of iron increased from 1.81 to 8.92% (**Table** **1**). On the other hand, copper, which is absent in natural zeolite, was incorporated by ≈5% due to ion exchange (Table 1). It can be seen that the content of elements other than silicon and aluminum increases by around 10% after ion exchange as compared to the native material (NZ). This suggests that the treatment is effective in localizing the main metal content at extra‐framework exchange sites. Due to their small amount, the metal content outside these sites seems to be dispersed over the whole material, as it is not observed in the SEM micrographs (Figure 1). When the characterization by DR‐UV–Vis is discussed below, the presence of nanoaggregates of these metals in the form of oxides or oxohydroxides will be demonstrated, which may explain the deviation of the excess metal content with respect to the exchange capacity. It was also demonstrated that Fe and Cu cations exchange with Na cations during the photo‐Fenton disinfection experiments, as will be discussed later.

**Table 1:** Elemental chemical composition (at. %) determined by EDX of NZ and the prepared catalysts before (NZ–Fe and NZ–Cu) and after (NZ–Fe and NZ–Cu post vis) photo‐Fenton tests.

Sample	Si	Al	Na	K	Ca	Mg	Fe	Cu	Cl
NZ	73.5 ± 1.5	14.0 ± 0.3	2.8 ± 0.1	1.95 ± 0.06	5.7 ± 0.1	0.14 ± 0.03	**1.81 **± 0.07	0.0	0.0
NZ–Fe	72.5 ±1.5	15.6 ± 0.4	0.0	0.54 ± 0.04	0.81 ± 0.04	0.43 ± 0.03	**8.9 **± 0.2	0.0	0.0
NZ–Fe post vis	73.2 ± 1.3	14.7 ± 0.3	**3.5** ± 0.1	0.0	0.0	0.0	**6.1 **± 0.2	0.0	**2.4 **± 0.1
NZ–Cu	76.1 ± 1.3	15.5 ± 0.3	0.0	0.73 ± 0.04	0.66 ± 0.04	0.0	2.01 ± 0.07	**4.9 **± 0.1	0.0
NZ–Cu post vis	71.8 ± 1.2	13.9 ± 0.3	**8.2** ± 0.2	0.63 ± 0.04	0.57 ± 0.03	0.0	1.40 ± 0.05	**1.16 **± 0.05	**2.3 **± 0.1

**Figure 2:**
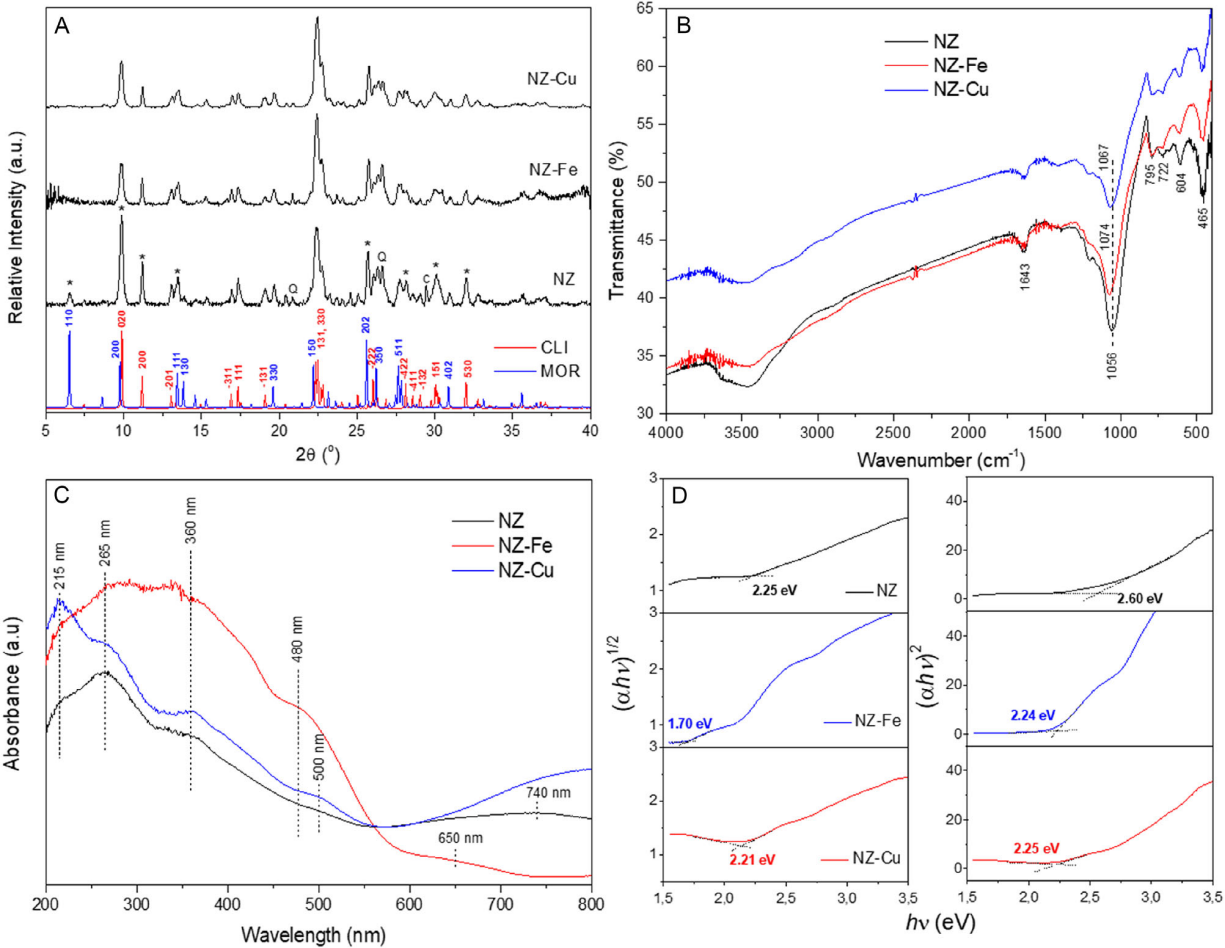
XRD powder patterns A), FTIR spectra B), UV–vis diffuse reflectance spectra C) and tauc plots for n exponents of 1/2 and 2 D) of natural zeolite (NZ) and samples exchanged with iron (NZ–Fe) and copper (NZ–Fu). Simulated XRD patterns of clinoptilolite (CLI) and mordenite (MOR) reported by the International Zeolite Association, are also shown for comparison. In the NZ pattern, peaks corresponding to the zeolitic phases whose relative intensity changes are indicated with an asterisk, while those corresponding to quartz and calcite are denoted by the letters Q and C, respectively.

The normalized X‐ray diffraction (XRD) patterns of the parent natural zeolite (NZ) and the Fe and Cu exchanged samples (NZ–Fe and NZ–Cu) are shown in **Figure** **2**A. The NZ pattern shows diffraction peaks at 2*θ *= 9.86°, 11.20°, 13.04°, 16.94°, 17.36°, 19.08°, 22.36°, 22.40°, 26.02°, 28.16°, 30.04°, and 32.00°, corresponding to the (020), (200), (−201), (−311), (111), (−131), (131), (330), (−222), (−422), (151), and (530) planes of clinoptilolite, respectively. This confirms that clinoptilolite is the main zeolitic phase in the mineral. Less intense diffraction peaks are also observed at 2*θ* values of 6.52°, 14.48°, 13.88°, 19.66°, 25.7°, 26.32°, 27.72° and 30.92°, corresponding to the (110), (111), (130), (330), (202), (350), (511) and (402) planes of mordenite. This indicates the mineral's lowest mordenite content, which is consistent with previous reports.^[^ ^32^ ^]^ The NZ sample was also found to contain quartz (peaks at 20.87° and 26.6°) and calcite (peak at 29.44°). It can be verified that the method used to obtain the samples enriched in iron and copper does not affect the zeolitic structure. Only variations in the relative intensities of some clinoptilolite and mordenite reflections are observed due to changes in the occupancy, nature, and population of the extra‐framework cationic sites.^[^ ^31^, ^33^ ^]^ The same behavior was observed in the zeolite patterns after the photo‐Fenton tests using visible and UVA light (see Figure S3, Supporting Information).

The Fourier transform infrared spectroscopy (FTIR) spectra of the zeolitic samples are shown in Figure 2B. The most intense band at 1056 cm^−1^ in NZ shifts to 1067 and 1074 cm^−1^ in NZ–Cu and NZ–Fe, respectively. This band is associated with external tetrahedral asymmetric stretching, and its shift to a higher wavenumber could be due to an average increase in the T—O bond strength. The Al—O tetrahedral bond is weaker than Si—O bond; therefore, differences in the Si/Al ratio of the zeolite can modify the frequency of the vibration. The observed shift to higher wavenumber could be due to some dealumination of NZ during the procedures carried out to obtain the exchanged samples, mainly during the obtaining of the acid form. The band shift may also be related to the ion exchange process involving iron and copper cations. While some authors have reported no significant changes in the FTIR spectrum of clinoptilolite due to exchange with transition metals,^[^ ^34^, ^35^ ^]^ others have observed a shift in the band depending on the nature of the cation (its charge and radius). For instance, Wan et al.^[^ ^36^ ^]^ observed this band shift from 1044 cm^−1^ in a synthetic clinoptilolite to 1055 cm^−1^ in iron‐exchanged clinoptilolite. In other words, the observed shift in our case may be due to a combination of dealumination of the lattice and ion exchange. On the other hand, no significant changes are observed in the spectra of the samples after being used in disinfection tests (see Figure S4, Supporting Information).

Figure 2C shows the diffuse reflectance UV–vis spectra of the initial zeolite and the samples exchanged with iron and copper cations. The NZ spectrum shows four bands at 265, 360, 500, and 740 nm. Synthetic clinoptilolite does not absorb for *λ *> 300 nm; therefore, the observed bands above this value are due to impurities present in the mineral.^[^ ^37^ ^]^ The 265 nm band corresponds to oxygen bound to the aluminum framework, and has also been assigned to the p‐d charge transfer transition between the framework's oxygen and the iron cation.^[^ ^38^ ^]^ The 360 nm band is assigned to octahedral Fe^3+^ in small Fe_2_O_3_ clusters, while the 500 nm band is associated with large Fe_2_O_3_ particles.^[^ ^39^, ^40^ ^]^ It has been reported that the band around 740 nm may be due to Fe^2+^ transitions or Fe^2+^‐Fe^3+^ intervalence charge transfer.^[^ ^41^, ^42^ ^]^ The presence of iron in natural zeolite (see Table 1) may explain the high absorption in the UV region and the presence of absorption bands in the visible region. After the iron exchange process (sample NZ–Fe), the absorbance of all bands increases, and the bands at 500 and 740 nm shift to lower wavelength values (480 and 650 nm, respectively).

In the NZ–Cu sample, the band at 215 nm is associated with a charge–transfer complex, due to the interaction between Cu^2+^ ions and ligand water molecules and with the zeolite framework.^[^ ^43^ ^]^ The band at 260 nm corresponds to the overlapping of oxygen bonded to framework aluminum and Cu^2+^ species interacting with oxygen from the zeolite structure. On the other hand, the bands in the 300–500 nm wavelength range correspond to the presence of copper in the form of Cu_*x*_O.^[^ ^43^ ^]^ Finally, the broad band at around 800 nm can be attributed to Cu^2+^ cations in octahedral coordination.^[^ ^44^, ^45^ ^]^

The bandgap energy (Eg) of the samples was estimated using the Tauc plot method, in accordance with the procedure reported by Makula et al.^[^ ^46^ ^]^ Figure 2D shows the graphs for the allowed direct (*n* = ½) and allowed indirect (*n* = 2) transitions for each sample, while Table S1 summarized the Eg values. A significant reduction in the bandgap of NZ with respect to the theoretical Eg value of 5.593 eV reported for an idealized clinoptilolite is observed.^[^ ^47^ ^]^ This is due to the presence of iron‐phases impurities in the mineral, as mentioned above. The incorporation of iron and copper into NZ reduces the bandgap energy of the catalysts for the indirect transition. This may be due to the inhibition of the electron–hole recombination process by the introduction of trap levels between the valence and conduction bands.^[^ ^48^ ^]^ Recent work has reported Eg values ranging from 2.08 eV to 1.88 eV (*n *= ½) and from 3.08 to 2.44 (*n* = 2) for different charged iron oxide clinoptilolite composites, with FeO content ranging from 4.1% to 13%.^[^ ^49^ ^]^ Additionally, Nippes et al*.* recently synthesized a heterogeneous solid iron catalyst with commercial zeolite Y, obtaining an estimated bandgap energy value of 2.95 eV.^[^ ^50^ ^]^ All of these Eg values are higher than those obtained for the NZ–Fe sample prepared by ion exchange (see Figure 1D). Amiri and Nezamzadeh‐Ejhieh^[^ ^51^ ^]^ reported a bandgap of 2.8 eV for CuO supported on clinoptilolite, which is higher than the bandgap energy of bulk CuO (1.2–1.5 eV). However, the authors found that supported CuO shows improved photocatalytic activity. Zhang et al*.* ^[^ ^52^ ^]^ also calculated a bandgap of 2.16 eV for a synthesized Cu‐based zeolite (Cu0 CuZ) containing copper and copper oxide nanoparticles and sodium as exchangeable cation. Our results confirm the optical properties of NZ–Fe and NZ–Cu, since the estimated bandgap energy values guarantee their ability to absorb light in the visible region, allowing them to be used effectively under solar radiation.

### 2.2 *E. coli* Inactivation by NZ–Fe/Cu & Vis—Assisted Photo‐Fenton: Reusability and Kinetics

**Figure 3:**
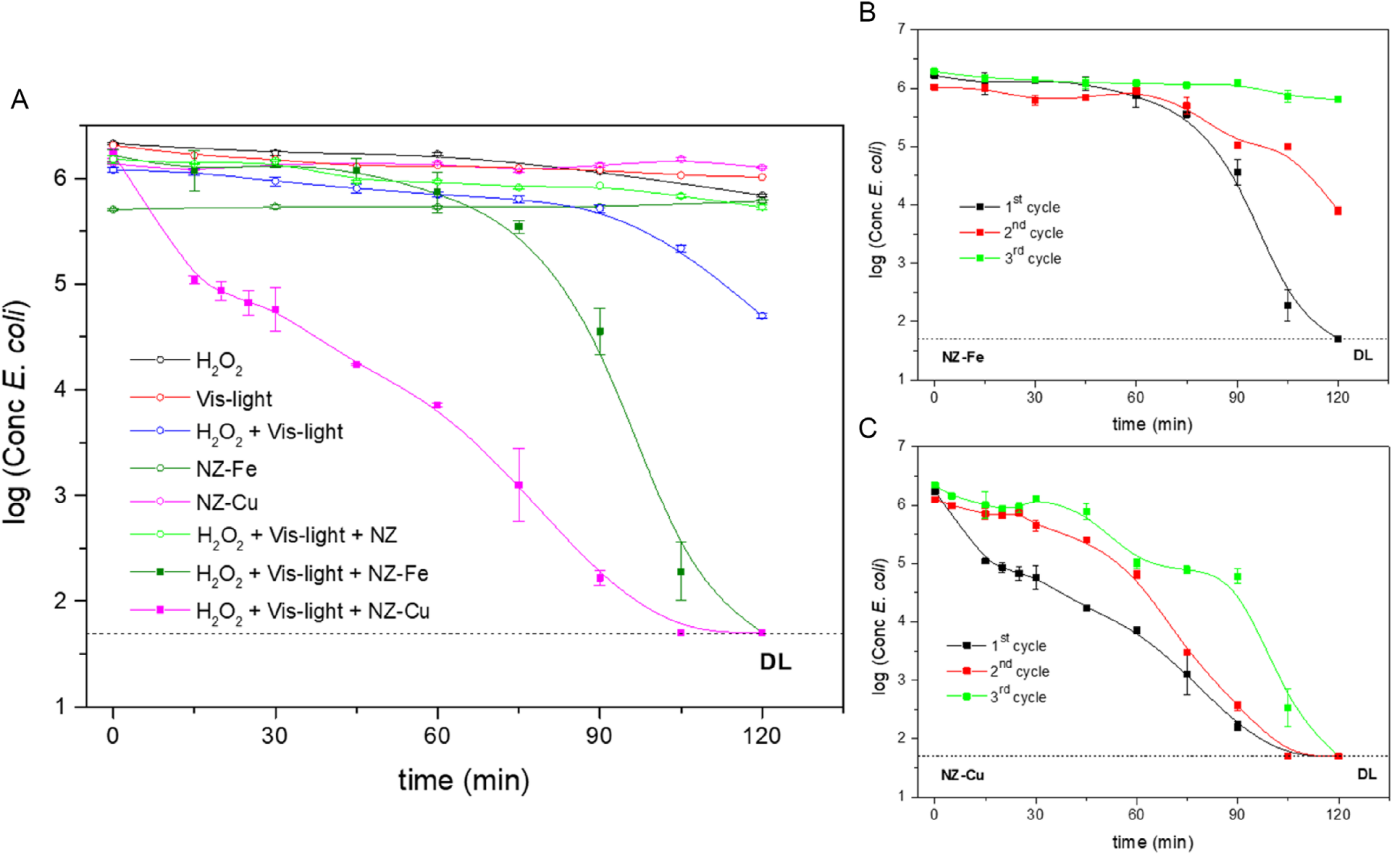
Inactivation of *E. coli* A) and consecutive cycles of *E. coli* inactivation B and C) by photo‐Fenton with visible light using the zeolitic catalysts. The error bars represent the standard deviation, which was calculated based on three independent counts of bacterial colonies. DL (detection limit) = 50 CFU/mL.

**Figure** **3**A shows the results of the photo‐Fenton inactivation of *E. coli* using the zeolitic catalysts under visible light. As the control assays demonstrate, NZ–Fe and NZ–Cu zeolites showed no bacterial inactivation in the absence of peroxide and light. Some literature reports refer to the use of copper‐exchanged zeolites as a microbicide. However, these studies involved a higher concentration of exchanged zeolite (1–10 g L^−1^) and longer contact times (3–24 h).^[^ ^11^ ^,^ ^53^, ^54^, ^55^, ^56^ ^]^ For instance, Milenkovic et al*.* ^[^ ^11^ ^]^ evaluated the microbicidal activity of natural clinoptilolite and zeolite A exchanged with Ag, Cu, and Zn against *E. coli*. They confirmed that 100% disinfection was achieved in a real water medium within 6 h and in commercial spring water within 3 h using copper‐exchanged clinoptilolite. The reusability of the bactericide zeolites was not considered in the study. In our study, however, under the employed conditions, the Cu^2+^ ion exchanged in the zeolite was not responsible for the inactivation of *E. coli*, as the bacteria were viable (culturable) for the entire period of the experiment (Figure 3A). Also, there is no bacterial inactivation when only visible light (or UVA; Figure S5, Supporting Information) is applied. This result is consistent with previous studies,^[^ ^57^ ^]^ which found that the visible light‐induced inactivation of this bacterium was negligible independently of the applied irradiance (50–400 W m^−2^). As expected, the addition of H_2_O_2_ did not reduce the bacterial population. Giannaskis et al*.*,^[^ ^58^ ^]^ found two main categories of H_2_O_2_ concentrations: a low concentration range (1–3 mM), where internal damage occurs, and a high concentration range (>20 mM), where external damage occurs due to the oxidizing action. In this study, an H_2_O_2_ concentration of 100 ppm (2.9 mM) was added to the system, placing it within the low concentration range, so the oxidant addition was not expected to induce cell damage. However, the combination of light and H_2_O_2_ caused a slight inactivation (1‐log) at 120 min. In the presence of UV and visible light, a substantial change must occur. Several authors have demonstrated that small amounts of H_2_O_2_ (0.015–1.5 mM) and solar radiation have a synergistic and lethal effect on resistant water microorganisms. For instance, fungal spores have been successfully inactivated in surface water,^[^ ^59^ ^]^ antimicrobial‐resistant *E. coli* in wastewater,^[^ ^60^ ^]^ total coliforms, *E. coli*, *Salmonella spp.*, and *Enterococcus spp.* in real wastewater,^[^ ^61^ ^]^ and multidrug‐resistant *Pseudomonas aeruginosa* in urban wastewater.^[^ ^62^ ^]^

This synergistic effect can be explained by the fact that the cell is first damaged by hydrogen peroxide's oxidative attack on the cell membrane. This initiates a chain of lipid peroxidation, increasing the membrane's permeability and compromising the viability of the bacteria. On the other hand, the cell partially damaged by this oxidative attack is subjected to photo‐absorption (UV and visible radiation), which produces greater bacterial inactivation.^[^ ^63^ ^]^ Complete inactivation occurs when UVA is applied in combination with H_2_O_2_ (Figure S5, Supporting Information). This could be due to the fact that the enzymes with antioxidant activity can be inactivated under UVA radiation, enabling the intracellular Fenton reaction to begin. Therefore, H_2_O_2_ penetration and UVA exposure weaken bacterial defenses, leading to greater inactivation.^[^ ^64^ ^]^

When both zeolitic catalysts are used in visible photo‐Fenton assays, complete bacterial inactivation (down to the detection limit of the method used) is achieved within 2 h (see Figure 3A). In the photocatalytic disinfection mechanism, it has been reported that reactive oxygen species (ROS) generated upon irradiation of the catalyst cause bacterial inactivation by partially breaking down of the external membrane, allowing the ROS to reach the cell wall and cytoplasmic membrane and resulting in cell lysis. Thus, bacteria are inactivated by the cumulative effects of repeated attacks by ROS on the cell wall‐membrane system.^[^ ^65^ ^]^ Furthermore, no bacterial regrowth occurred within 24 h (data not shown), suggesting that internal repair mechanisms such as enzyme induction (peroxidase, catalase, etc.) for DNA repair by scavenging ROS do not occur. This indicates that photo‐Fenton treatment with both materials causes irreversible damage to bacteria. On the other hand, when natural clinoptilolite is added without iron or copper exchange, but in the presence of hydrogen peroxide and radiation, bacterial inactivation is negligible. This demonstrates that the photo‐Fenton reaction only occurs when the exchanged clinoptilolites are used and that natural zeolite exhibits no photocatalytic activity under the tested conditions, as previously reported.^[^ ^30^ ^]^ The disinfection curves take different forms depending on the exchanged metal cation (Figure 3A). Three regions can be identified in the NZ–Fe zeolite plot: an initial delay, a smooth decay up to 75 min, and a linear decrease with higher velocity toward the end of the experiment. This behavior suggests an initial superficial mechanism that may be due to sub‐lethal damage to the bacterial cell membrane. For the NZ–Cu catalyst, however, the initial delay is not observed. Instead, an almost linear decrease in *E. coli* concentration as a function of time is observed from the beginning, with the detection limit being reached at a slightly shorter time. This catalyst appears to cause more severe damage to the cell membrane from the start of irradiation, leading to faster inactivation kinetics. Results from control performed using this zeolite in the absence of hydrogen peroxide and light demonstrate that NZ–Cu alone does not affect bacterial viability, as previously mentioned. Therefore, the difference may be due to the volumetric rate and the type of ROS generated in each case.

Three consecutive disinfection cycles were performed using the same catalyst in order to evaluate its reusability. The results are shown in Figures 3B and 3C. When the iron catalyst was used in a second cycle, the shape of the curve was similar, and bacterial inactivation was observed, but the detection limit was not reached within two hours of the test. In a third cycle, practically no inactivation activity was observed. The copper catalyst remained effective up to three photo‐Fenton cycles, reaching the detection limit in all cases, although at a slightly longer time. The fact that complete inactivation was achieved after the third cycle is a very good result in terms of reducing the cost of catalyst application. Interestingly, the amount of metal cation that leaves the catalysts after the three disinfection cycles is 31% for iron and 76% for copper (see Table 1), which could be attributed to the higher solubility of the Cu (II) ion than the Fe (III) species in water at neutral pH.^[^ ^66^ ^]^ This demonstrates that even a very small amount of copper is enough to produce the active species required for bacterial inactivation with practically the same effectiveness.

**Figure 4:**
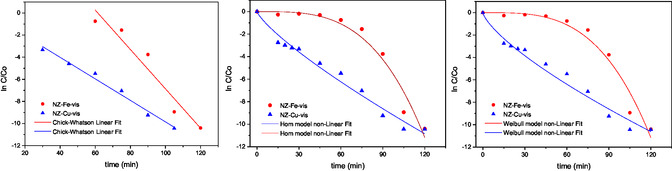
Fitting of the experimental data to the Chick–Watson model (linear portion of the disinfection curves) and the Hom and Weibull models (complete data).

The experimental kinetic data were analyzed according to the Chick–Watson first‐order equation, the Weibull model, and the Hom model. The results are presented in **Table** **2** and **Figure** **4**. As previously mentioned, when the NZ–Fe catalyst is used, the inactivation curve exhibits an initial delay, commonly referred to as a shoulder. This shoulder may be due to the time it takes for the bacterial cells to show the effect of the stress‐induced damage. Subsequently, this phase is followed by a linear decay until the detection limit is reached. For this sample, the experimental data best fit the Weibull model, which, unlike the Chick–Watson model, considers the delay in disinfection. In the case of the NZ–Cu catalyst, a decrease in bacterial concentration is observed practically from the beginning of the photo‐Fenton process, and consequently, the experimental data fit the Chick–Watson model better.

**Table 2:** Kinetic parameters of the experimental data fitted according to different models.

Model/catalyst	NZ–Fe	NZ–Cu
Chick–Watson model	*K*_max_ = 0.18 ± 0.02 min^−1^ R^2^ = 0.9143	*K*_max_ = 0.097 ± 0.006 min^−1^ R^2 ^= **0.9800**
Hom model	K′ = 1 × 10^−6 ^± 2 × 10^−6 ^min^−h^ *h* = 3.4 ± 0.4 R^2^ = 0.9571	K′ = 0.24 ± 0.05 min^−h^ *h* = 0.80 ± 0.06 R^2^ = 0.9759
Weibull model	Δ = 58 ± 5 min^−1^ *p* = 3.4 ± 0.5 R^2^ = **0.9572**	Δ = 4.8 ± 0.8 min^−1^ *p* = 0.73 ± 0.04 R^2^ = 0.9774

### 2.3 Hydroxyl Radical Detection

**Figure 5:**
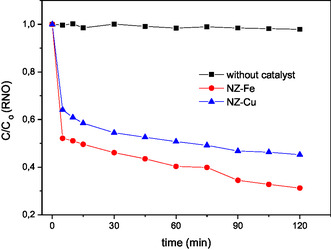
Degradation of p‐nitrosodimethylaniline by hydroxyl radicals generated with visible light and H_2_O_2_, in the absence and presence of catalysts.

To further study the behavior observed in the inactivation kinetics of *E. coli*, the generation of the hydroxyl radical was evaluated using p‐nitrosodimethylaniline bleaching (**Figure** **5**).

The amount of hydroxyl radicals generated in the absence of a catalyst is very low, which is consistent with the behavior observed in the corresponding control (see Figure 3A). While the curves for both catalysts are similar in shape, NZ–Fe showed a slightly higher hydroxyl radical production than NZ–Cu (see Figure 5). This behavior is not correlated with the results of *E. coli* photo‐Fenton disinfection assays using the NZ–Fe and NZ–Cu catalysts, where NZ–Cu showed more effectiveness and a rapid degradation kinetics (see Figure 3). This suggests that ^•^OH radicals are not the only mechanism acting in the photo‐disinfection process, and other species must be active in inactivating *E. coli*. For example, it has been reported that, at a pH close to neutrality (at which the tests in this work were conducted) and in the presence of hydrogen peroxide, high‐valent cupryl ion (Cu (III)) is an active specie produced by the reaction of Cu (I) with H_2_O_2_ via a two‐electron transfer.^[^ ^67^, ^68^ ^]^ Likewise, another high‐valent species in the case of iron (ferryl ion; Fe (IV)) can also act at neutral pH as an oxidizing species with high efficiency.^[^ ^68^, ^69^ ^]^ Thus, the presence of such species could be possible in this work, although additional studies are required to confirm it.

### 2.4 *E. coli* Inactivation Studies with Leachate of the Catalysts

**Figure 6:**
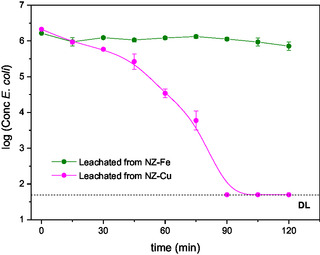
Inactivation of *E. coli* by photo‐Fenton under visible light using the leachate of the catalysts. The error bars represent the standard deviation, which was calculated based on three independent counts of bacterial colonies. DL (detection limit) = 50 CFU/mL.

To determine whether the metal released from the catalyst is responsible or plays a role in the inactivation of *E. coli*, experiments were conducted in which water from the disinfection test (after sterilization) was reinjected with bacteria, and a new photo‐Fenton process was performed without adding the catalyst. The results show that the iron leached from the catalyst does not significantly inactivate the bacteria (**Figure** **6**). This indicates that only the iron exchanged in the zeolite participates in the production of ROS. In the case of copper, both the exchanged cation and the leached cation participate in the photocatalytic process. However, it should be noted that the *E. coli* inactivation mechanism differs when copper is in solution, as an initial delay in the curve is observed, although the detection limit is reached in a slightly shorter time. Finally, it is important to note that even though copper is lost during the photo‐Fenton process, the catalyst's lifetime is not compromised for at least three treatment cycles, as discussed above.

## 3 Conclusions

This study demonstrates that natural zeolite clinoptilolite, when exchanged with Fe or Cu (NZ–Fe and NZ–Cu), is an effective heterogeneous photo‐Fenton catalyst for bacterial inactivation, without the need for additional co‐catalysts. The performance of this modified zeolite was validated by its ability to inactivate *E. coli* in water, reaching the detection limit without any observed bacterial regrowth. This demonstrates for the first time the high efficiency of this type of catalyst for water disinfection by heterogeneous photo‐Fenton reactions. DR‐UV–vis measurements showed that both materials have the optimal bandgap energy for absorbing visible light, ensuring high efficiency under solar radiation. Although NZ–Fe produced more hydroxyl radicals and less leachate, NZ–Cu showed faster kinetics and superior reusability over three disinfection cycles. This is likely due to a higher copper leachate and the potential contribution of additional bactericidal species beyond hydroxyl radicals. In conclusion, natural clinoptilolite incorporating metals could be employed as sustainable catalysts for heterogeneous photo‐Fenton‐based disinfection, particularly in water treatment applications, due to their low cost and advantageous physicochemical properties. Further experimental research will explore the treatment of real contaminated water from both natural and anthropogenic sources, and provide an economic assessment of the entire treatment compared with standard AOPs.

## 4 Experimental Section

4.1

4.1.1

##### Preparation of the Zeolitic Materials

The starting material used was the natural zeolite from the Tasajeras deposit (Cuba). The mineral was sieved and the fraction with particle size between 40 and 90 µm was purified by successive washings with distilled water at room and boiling temperatures to remove accompanying phases. The purified zeolite was denoted as NZ. Previous analysis of the XRD pattern of this purified mineral using a mixture of crystallographic and mineralogy methods showed that it is composed of 70% clinoptilolite, 5% mordenite, 5% anorthite, and 10% quartz.^[^ ^32^ ^]^

An acid sample of NZ was prepared by ion exchange with ammonium chloride (highly favored in clinoptilolite), followed by calcination at 450 °C for 5 h. Iron‐enriched (NZ–Fe) and copper‐enriched (NZ–Cu) samples were prepared by ion exchange of the acid NZ, with the aim of achieving a high degree of exchange.^[^ ^31^ ^]^ In all the ion exchange processes, a concentration of 0.5 M of the corresponding salt (NH_4_Cl, FeSO_4_, and Cu (NO_3_)_2_) was used, performing three exchange cycles of 4 h at a temperature of 80 °C, and a solid/liquid ratio of 1g 10 mL. After the last exchange, the samples were washed repeatedly with distilled water to remove excess salt and allowed to dry in an oven at 100 °C. The prepared materials and also those after the photocatalytic tests were characterized by using XRD, FTIR, UV–vis spectroscopy (UV–vis), and scanning electron microscopy (SEM/EDX). Details are shown in the Supporting Information.

##### *E. coli* Inactivation by Zeolites Assisted Photo‐Fenton

*E. coli* strain K12 was used as a model to evaluate the catalytic activity of the zeolitic materials. The preparation of the microorganism stock and the inoculum are described in the Supporting Information. For the photo‐Fenton tests, saline solution (0.9% w/v NaCl) with an initial concentration of *E. coli* of 10^6^ CFU/mL, 100 ppm (2.9 mM) of H_2_O_2_, and 1 g/L of the zeolitic catalyst were used at a pH close to neutrality (≈6 without external adjustment). A double‐jacketed glass 1L reactor with water recirculation was used for testing (Figure S1, Supporting Information). It was operated at a constant temperature of 25 °C and with magnetic stirring at 500 rpm. The reactor was covered with aluminum foil to improve and redistribute light inside the reactor, and radiation was delivered by a submerged visible light lamp (410–710 nm/9 W m^−2^, submerged length 21 cm). Experiments were also carried out using a UVA submerged lamp (374 nm/29 W m^−2^) (results in Supporting information). The emission spectra of the lamps are shown in Figure S2, Supporting Information.

The reactor was filled with the saline solution, and a control sample was taken to verify the absence of microorganisms in both, reactor and the solution. An overnight *E. coli* culture in LB (10^9^ CFU/mL) was centrifuged and washed to eliminate residual culture medium and used to inoculate the reactor. Then, after stirring for homogenization, a sample was collected to determine the initial concentration of bacteria for each experiment. A “dark‐control” sample was taken and kept in the dark at room temperature and plated twice, at the start and end of the experiment, to confirm the correct viability of the bacteria in the saline solution during the tests. Subsequently, zeolitic material and hydrogen peroxide were added in the dark. After homogenization of the system, the reactor was exposed to the radiation source, and samples were taken at predetermined times for 2 h. To quench the action of H_2_O_2_ over bacteria during the time interval between sampling and plating, the sample was mixed with the required amount of catalase solution. The experiments were done in triplicate; the results did not show significant statistical variations (confidence level > 95%). Once each treatment was finished (at 2 h), a “final‐control” sample was reserved and plated twice at the sampling time and 24 h after to evaluate any potential bacterial regrowth. Solution samples taken during the experiment were enumerated using the standard plate counting method through tenfold serial dilutions in LB. Colonies were counted after 24 h incubation at 37 °C. This procedure was carried out in triplicate for each sample. The detection limit was 50 CFU/mL. After each treatment, the reactor was thoroughly cleaned with sodium hypochlorite 50% v/v and then washed three times with distilled water. Additionally, all materials and prepared solutions were sterilized by autoclaving at 1 bar, 121 °C for 20 min.

To study the reuse of the catalysts, three consecutive cycles of disinfection of the microorganism were carried out with the same zeolitic material, using the procedure and conditions previously described. Following the initial cycle, the catalyst was separated via decantation and filtration. It was then washed with distilled water and dried at 100 °C for 24 h. Prior to the next cycle, the catalyst was weighed, and the solution volume was adjusted to maintain a constant catalyst‐to‐solution ratio of 1 g L^−1^.

To evaluate the disinfection process, the kinetics of the bacterial inactivation were studied. Details are accessible in the Supporting Information. In connection with an in‐depth understanding of the catalytic processes, tests were conducted to see in which extent the hydroxyl radicals are the key elements and also the metal ions leached into the solutions (see details in the Supporting Information).

## Conflict of Interest

The authors declare no conflicts of interest.

## Supporting information

Supplementary Material

## Data Availability

The data that support the findings of this study are available from the corresponding author upon reasonable request.
