# Supplementary material for: Cu or Fe‐Exchanged Natural Clinoptilolite as Sustainable Light‐Assisted Catalyst for Water Disinfection at Near Neutral pH
Source: Chempluschem. 2025 Sep 18;90(11):e202500225. doi: 10.1002/cplu.202500225 (PMC12605726; doi:10.1002/cplu.202500225)
Supplement: Supplementary file 1 — Supplementary Material [file CPLU-90-e202500225-s001.pdf]

Supporting Information content

1. Materials and Methods

1.1. Chemicals Reagents

1.2. Characterization of zeolites

1.3. Preparation of the microorganism stock

1.4. Preparation of the inoculum

1.5. Experimental Setup

Figure S1: Photography of the experimental set-up.

Figure S2: Emission spectrum for (a) UV light lamp, (b) visible light lamp.

1. 6. Inactivation kinetics

1. 7. Hydroxyl radical detection

1.8. *E. coli* inactivation studies with leachate of the catalysts

2. Results

Figure S3: XRD powder patterns of the starting zeolite NZ, and the Fe (A) and Cu (B) exchanged samples before and after the disinfection assays using visible and UVA light. Peaks that change their relative intensity after treatments are indicated with an asterisk.

Figure S4: FT-IR spectra of the samples after the photo-Fenton assays using UVA and visible light.

Table 1S: Band gap energy (Eg) of the samples estimated using the Tauc plot method for n exponents of 1/2 and 2.

Figure S5. Inactivation of *E. coli* by photo-Fenton with UVA light using the zeolitic catalysts. DL (detection limit) = 50 CFU/mL.

1. Materials and Methods

1.1. Chemicals Reagents

Ammonium chloride (NH_4Cl, PanReac, >99%), iron (II) sulfate heptahydrate ($FeSO_4x7H_2O$, WVC, 99%) and copper (II) nitrate trihydrate ($Cu(NO_3)_2x3H_2O$, WVC, 99%) were used for the modification of the natural zeolite. Hydrogen peroxide (H_2O_2, PanReac, 30%) and catalase from bovine liver (Sigma-Aldrich, 2000-5000 units/mg protein) were used for the *E. coli* inactivation assays. Tryptone (Condalab), yeast extract (Condalab) and sodium chloride (NaCl, Labkem, > 99.9%) were employed to prepare Luria-Bertani (LB, 10 g/L tryptone, 5 g/L yeast extract and 5 g/L NaCl) culture medium.

1.2. Characterization of zeolites

Qualitative X-ray diffraction (XRD) analysis was used to ensure the structural stability of the zeolite when subjected to ion exchange and disinfection assays. The possible appearance of new phases and changes in the relative intensities were also evaluated. A Bruker D8 Discover diffractometer was used, using Cu Kα radiation (λ = 1.541838 Å). The 2θ range from 5 to 50° was swept at 2°/min in a continuous way.

Infrared spectroscopy analysis was performed using a JASCO 4100 Fourier Transform infrared spectrometer in the wavenumber interval of 400 – 4000 cm^{-1}. Samples were prepared using the KBr pressed-disk technique, with a 1% inclusion of the material to be analysed.

The optical properties of the samples were studied in the wavelength interval of 200 to 800 nm by UV-Vis spectroscopy, using an Agilent Cary 100/300 Series UV-Vis, equipped with an internal diffuse reflectance accessory (DRA) module.

The microstructure, morphology, and chemical composition of the samples were studied by scanning electron microscopy (SEM/EDX). A Hitachi S4800 SEM-FEG model microscope was used, which has a cold cathode field emission gun and voltage of 0.5-30 kV, resolution of 1 nm at 15 kV. It is equipped with a Bruker-X Flash-4010 EDX analyser with a resolution of 133 eV on the MnKα line, a slew window AP3.3 and a detector with a sample holder to work in transmission mode (STEM-in-SEM). Analysis was performed by mapping a selected representative area of each sample, with a counting time of 15 minutes.

1.3. Preparation of the microorganism stock

Cryopreserved vials were prepared from strains supplied by the CABD. For this, a Petri dish is streaked with LB medium and incubated under optimal conditions (24 h, 37 ºC, 180 rpm). Subsequently, an inoculum is prepared starting from the incubated plate in a sterile tube containing 5 mL of LB liquid medium. A control tube is also prepared. The tubes are incubated under optimal conditions on an orbital shaker. After incubation a cryopreservation vial is prepared with 300 µL of 50% glycerol and 700 µL of the inoculum, and stored ultrafrozen at -80 ºC. The estimated conservation period is approximately 6 months.

To obtain a stock plate, the previously prepared cryopreserved vial is used. The surface of the frozen strain is rub and spread in a plate with the appropriate medium for the microorganism. The plate is incubated under optimal conditions. Once incubated, another streak seeding is carried out from it in order to obtain a stock plate with isolated differentiated colonies. This re-seeding of the plate is repeated every 2 weeks in order to maintain maximum viability and homogeneity of the strain.

1.4. Preparation of the inoculum

An isolated colony is taken from the obtained stock plate (section 1.3) and is inoculated in a sterile tube with 5 mL of LB liquid medium, which is incubated under optimal conditions with constant agitation.

After incubation, the microorganism must be in the stationary phase of growth. From the inoculated tube, the biomass is recovered by centrifugation at 3000 rpm for 5 min. Subsequently, the pellet is washed three times with 5 mL of a 0.9 % w/v sodium chloride solution, and finally it is resuspended in 5 mL of saline solution to obtain an approximate concentration of 10^9 CFU/mL.

1.5. Experimental Setup

The experimental setup used is shown in Figure S1. Glasses containers in the right side of the reactors contain the zeolite samples. The lamp is introduced inside the reactor. Its spectral features are shown in Figure S2.

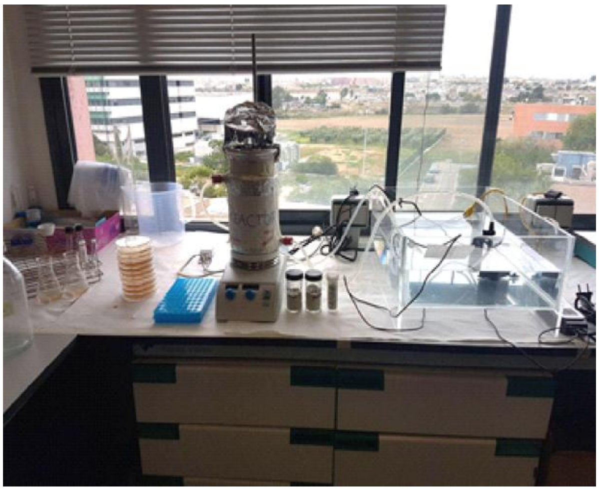

Figure S1: Photography of the experimental set-up

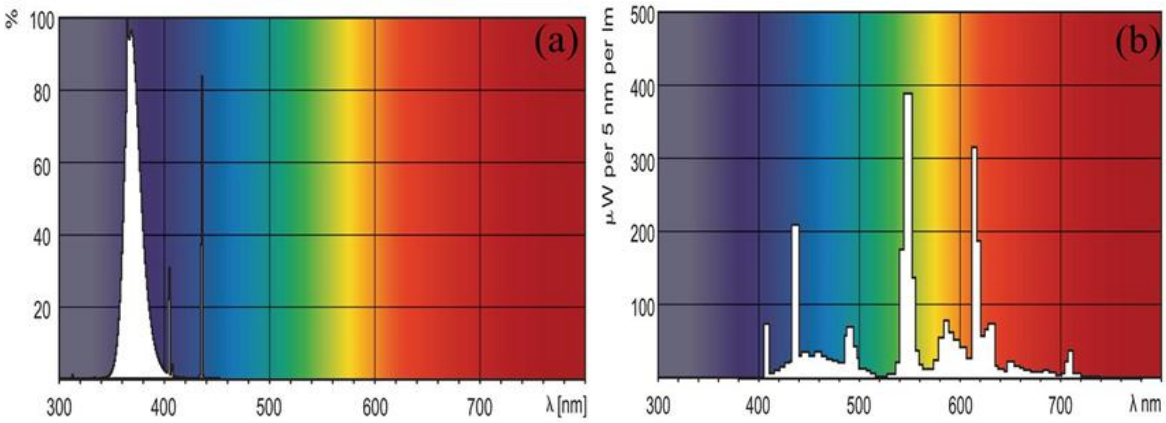

Figure S2: Emission spectrum for (a) UV light lamp, (b) visible light lamp

1.6. Inactivation kinetics

In order to study the characteristics of the disinfection process, the experimental data were fitted according to the Chick-Watson first order equation (Eq. 1), the Weibull model (Eq. 2) and the Hom model (Eq. 3).

$$\ln\left(\frac{C}{Co}\right) = -kt \quad (1)$$

$$\ln\left(\frac{C}{Co}\right) = \left(-\frac{t}{\Delta}\right)^{p} \quad (2)$$

$$\ln\left(\frac{C}{Co}\right) = -k^{\prime}t^{h} \quad (3)$$

where C/C_0 is the reduction in microorganism concentration; *k, Δ and k´* are the kinetic constants of disinfection of Chick-Watson, Weibull, and Hom, respectively; *p* and *h* are shape parameters, and *t* is the treatment time. In Weibull and Hom models, if the shape parameter is lower than 1, then the survival curve is concave; if the shape parameter is greater than 1 then it is convex, when the shape parameter is equal to 1 the curve simplifies to the Chick-Watson linear equation.

1.7. Hydroxyl radical detection

Reagent p-nitrosodimethylaniline (RNO), a specific hydroxyl radical scavenger, was used as a probe compound and a spin trap for detection of hydroxyl radicals using UV-Vis spectrometry. Zeolitic catalyst were suspended in a stirred solution containing RNO (17 µM) and the necessary amount of 30 % H_2O_2 to have a concentration of 100 ppm. The solution was maintained at 25 ^{o}C and irradiated with visible light. Samples were taken, centrifuged at 400 rpm, and RNO concentration was evaluated at 440 nm in a UV–Vis spectrometer. Control assay without zeolite were carried out.

1.8. *E. coli* inactivation studies with leachate of the catalysts

To study the effect of iron and copper cations leached from the catalysts to the medium on the inactivation of *E. coli*, tests with the water resulting from the photocatalytic experiment (where the bacterial detection limit was archived) were carried out. For this, the reactor was filled with the resulting water from photo-Fenton assays. This water was previously sterilised at 121 ^{o}C for 20 minutes. The same procedure described in the Experimental Section of the paper was followed, but without adding the zeolitic catalyst just using the existing water after removing the zeolitic materials and just adding the bacterial inoculum and the oxidizing agent.

2. Results

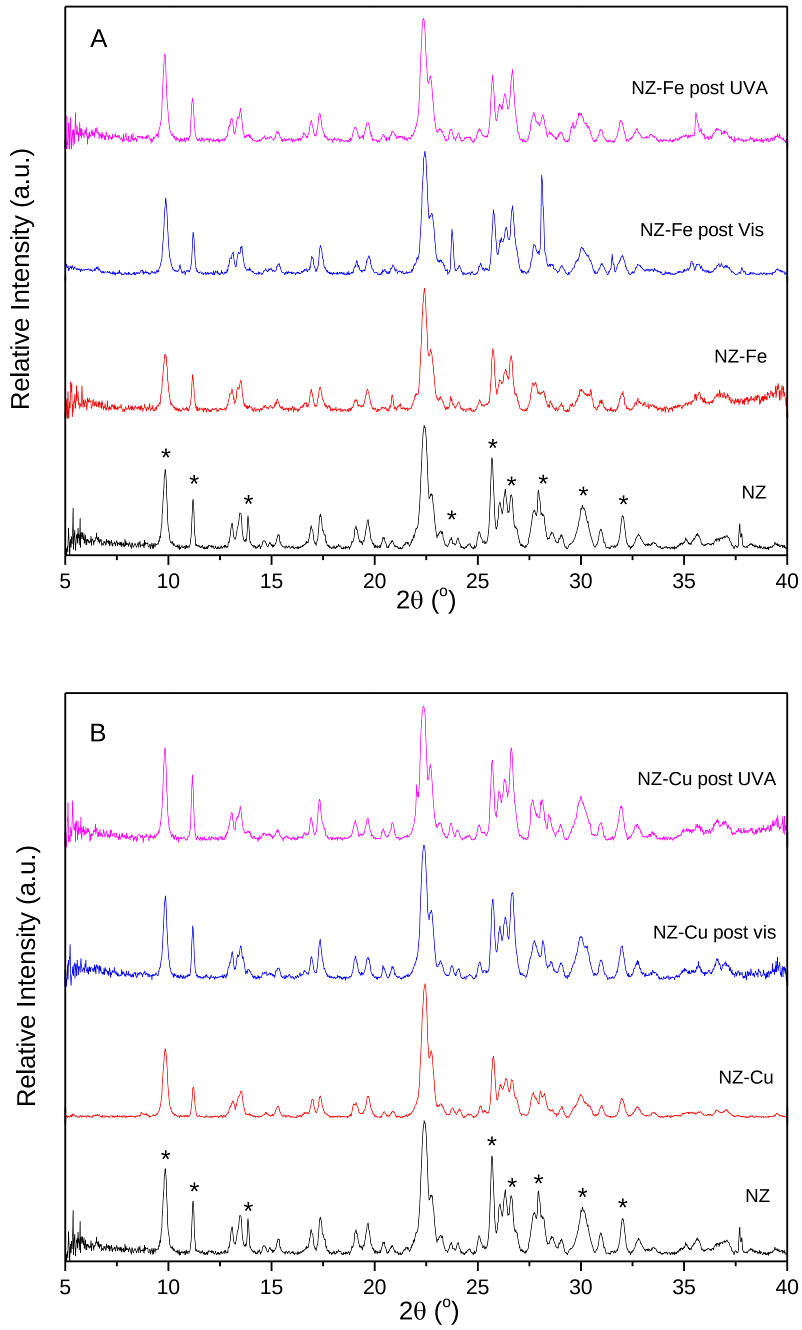

Figure S3: XRD powder patterns of the starting zeolite NZ, and the Fe (A) and Cu (B) exchanged samples before and after the disinfection assays using visible and UVA light. Peaks that change their relative intensity after treatments are indicated with an asterisk.

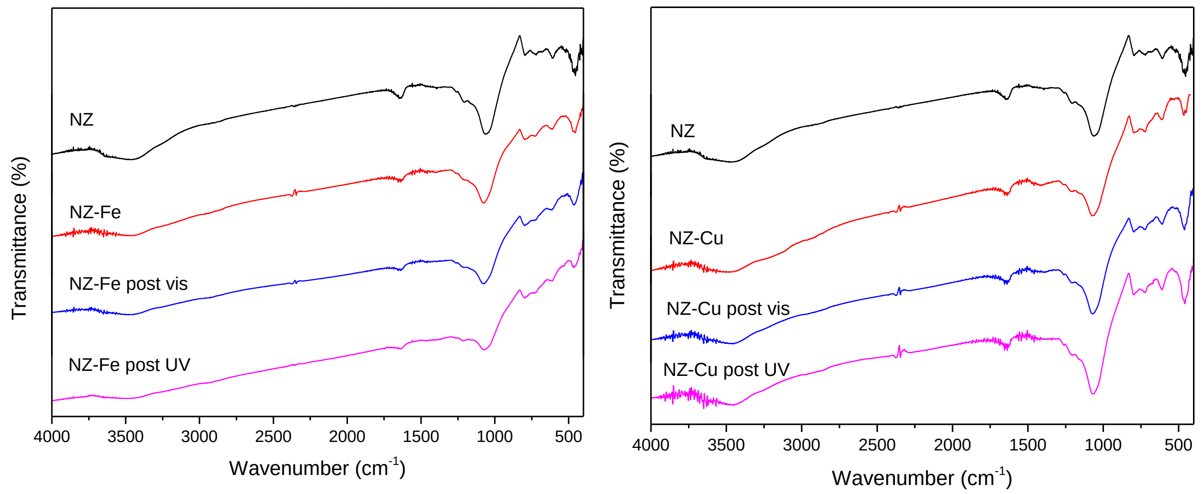

Figure S4: FT-IR spectra of the samples after the photo-Fenton assays using UVA and visible light.

Table 1S: Band gap energy (Eg) of the samples estimated using the Tauc plot method for n exponents of 1/2 and 2.

**Sample**	**Eg (eV)**	
	**n = 1/2**	**n = 2**
NZ	2.25	2.60
NZ-Fe	1.70	2.24
NZ-Cu	2.21	2.25

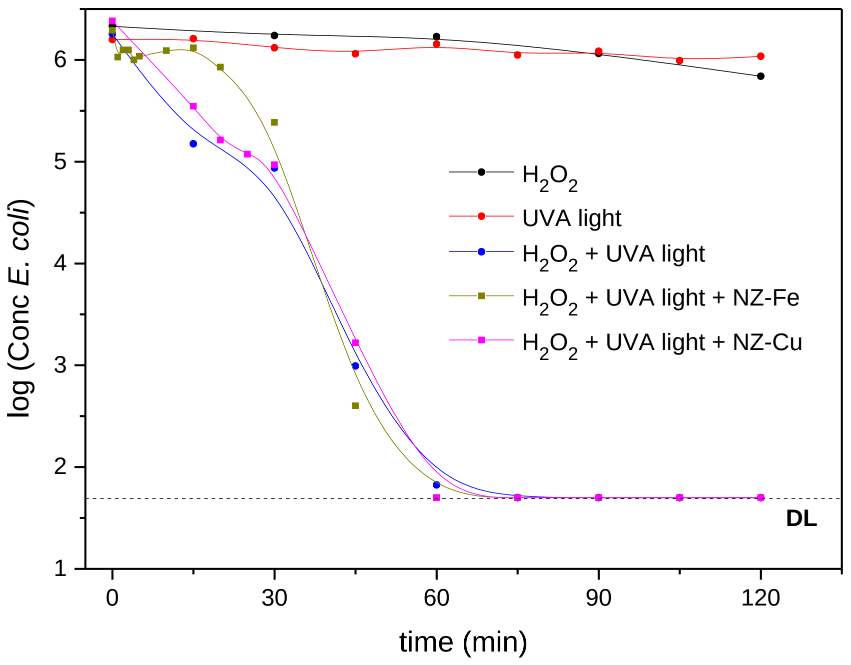

Figure S5. Inactivation of *E. coli* by photo-Fenton with UVA light using the zeolitic catalysts. DL (detection limit) = 50 CFU/mL.
